# Elevated Serum Total Bilirubin Might Indicate Poor Coronary Conditions for Unstable Angina Pectoris Patients beyond as a Cardiovascular Protector

**DOI:** 10.1155/2023/5532917

**Published:** 2023-09-05

**Authors:** Qi Liang, Yongjian Zhang, Jin Liang

**Affiliations:** ^1^Department of Cardiology, First Affiliated Hospital of Xi'an Jiaotong University, No. 277 Yanta Rd, Shaanxi, Xi'an 710061, China; ^2^Department of Medical Insurance, Xi'an Affiliated Hospital of the Shaanxi University of Chinese Medicine, China

## Abstract

**Backgrounds:**

Serum total bilirubin (STB) is recently more regarded as an antioxidant with vascular protective effects. However, we noticed that elevated STB appeared in unstable angina pectoris (UAP) patients with diffused coronary lesions. We aimed to explore STB's roles in UAP patients, which have not been reported by articles.

**Methods and Results:**

1120 UAP patients were retrospectively screened, and 296 patients were finally enrolled. They were grouped by Canadian Cardiovascular Society (CCS) angina grades. The synergy between PCI with TAXUS stent and cardiac surgery score (SYNTAX score) and corrected thrombolysis in myocardial infarction flow count (CTFC) were adopted to profile coronary features. The results showed that STB, mean platelet volume (MPV), hs-CRP, fasting blood glucose (FBG), red blood cell width (RDW), and CTFC elevated significantly in the CCS high-risk group. STB (*B* = 0.59, 95% CI: 0.39-0.74, *P* < 0.01) and MPV (*B* = 0.86, 95% CI: 0.42-1.31, *P* < 0.01) could indicate SYNTAX score changes for these patients. STB (≥21.7 *μ*mol/L) could even indicate a coronary slow flow condition (AUC: 0.88, 95% CI: 0.84-0.93, *P* < 0.01). Moreover, UAP patients with elevated STB had a lower event-free survival rate by the Kaplan-Meier curve. STB ≥21.7 *μ*mol/L could reflect a poor coronary flow status and indicate 1-year poor outcomes for these patients (HR: 2.01, 95% CI: 1.06-3.84, *P* < 0.01).

**Conclusion:**

Elevated STB in UAP patients has a close relationship with changes in SYNTAX score. STB (over 21.7 *μ*mol/L) could even indicate a coronary slow flow condition and poor outcomes for the UAP patients.

## 1. Introduction

Unstable angina pectoris (UAP) frequently present diffused coronary lesions and tend to progress into severe conditions [1]. In the clinical, coronary flow has close relationships with coronary lesions, and abnormal coronary flow reflects poor clinical conditions for UAP patients. In UAP patients with high-risk tendencies, oxidative stress and reactive oxidative species (ROS) could frequently be found. It was reported that oxidative stress and ROS attack the cardiovascular system and lead to the rapid progress of coronary lesions [2]. ROS is harmful to endothelial cells and atherosclerotic caps [3]. Due to their negative impacts on coronary vessels, oxidative stress and ROS attract significant attention recently. Having a close relationship with oxidative stress, STB in recent years is more regarded as an antioxidant with vessel protective effects [4–6]. However, we referred to articles and found that STB's roles were underdebated in diseases [7]. We also noticed that elevated STB had close relationships with poor conditions in UAP patients.

STB is catalyzed by heme degradation under the activation of heme oxygenase-1(HO-1). In addition to STB's antioxidative ability, elevated STB is also linked to poor clinical conditions. A meta-analysis showed that STB had a positive correlation with in-hospital cardiovascular mortality [8]. Based on our cases, we proposed that elevated STB might reflect oxidative burden added to the cardiovascular system. To explore STB's roles in UAP patients, we adopted the Synergy between PCI with TAXUS drug-eluting stent and Cardiac Surgery (SYNTAX) score to profile changes in coronary lesions. SYNTAX score is a widely accepted and convenient tool for the investigation of coronary atherosclerosis [9].

To depict coronary flow changes, corrected TIMI flow frame count (CTFC) was adopted. Due to the close relationships between clinical status and coronary blood flow, we proposed that elevated STB might reflect a poor coronary flow status for the UAP patients, which has not been reported. In clinical work, it is difficult to identify poor coronary blood flow through noninvasive methods, and the ones without typical chest pain might also be ignored. Being an easily acquired biochemistry molecular, STB might be promising and convenient in making clinical decisions for UAP patients. Due to these premises, we aimed to investigate the roles of STB in coronary changes and further explore the indicative effects of STB on clinical outcomes for UAP patients.

## 2. Methods

### 2.1. Study Population and Group Division

We retrospectively recorded the data of consecutive patients who went to our hospital from Jan 2019 to Dec 2021. 1120 UAP patients were screened. Finally, 296 patients who met the study inclusion and exclusion criteria were enrolled. This study was performed according to the Declaration of Helsinki and approved by the ethical committee of the First Affiliated Hospital of Xi'an Jiaotong University. Informed consent was obtained from the patients. The reasons for the excluded patients were shown in Supplementary Table 1. The basic features of the enrolled and excluded patients were listed in the Supplementary Table 2.

**Figure 1:**
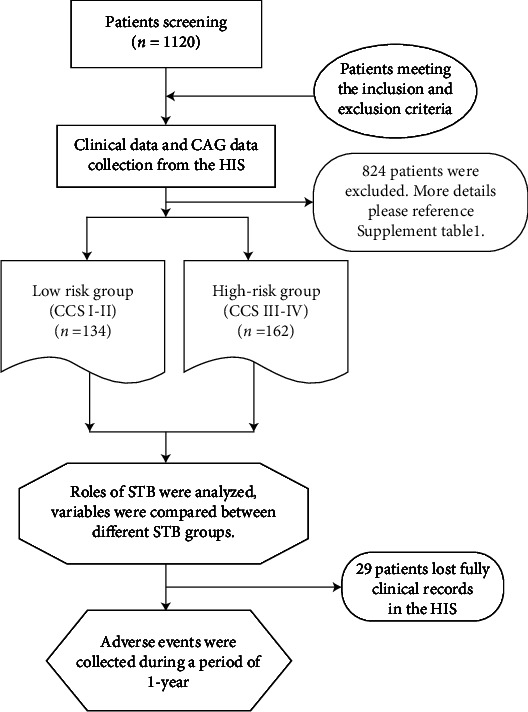
Flow chart of the study.

The patients were divided by risk grades according to the Canadian Cardiovascular Society (CCS) angina risk classification [10]. Clinical variables were compared between the CCS low-risk group (CCS I-II, *n* = 134) and the CCS high-risk group (CCS III-IV, *n* = 162). The flow chart of this study is shown in Figure 1.

### 2.2. Inclusion and Exclusion Criteria

Inclusion criteria are the following: (1) UAP patients were primarily screened. The diagnostic standard was based on the guideline of “Unstable angina pectoris and ST-segment elevation myocardial infarction: Guidelines for diagnosis and treatment” [11]. (2) CAG should be performed for rational diagnostic or therapeutic purposes as documented from HIS. (3) The patients enrolled should be willing to share their clinical data and signed informed consent.

Exclusion criteria are the following: (1) Patients aged ≤ 18 and ≥80 years old and physically weak were excluded. (2) Patients with contraindications for CAG, such as acute infectious disease, platelet count ≤ 20^∗^10^9^, or hypercoagulability status were excluded. (3) Patients with clinical conditions affecting the STB results, such as hepatobiliary disease, acute hepatitis, kidney dysfunction, and pancreas disease were excluded. (4) Patients with recurrent jaundice or jaundice history were excluded due to the possible Gilbert syndrome. (5) Patients with ALT ≥ 80 IU/L, AST ≥ 80 IU/L, and ALP ≥ 220 IU/L reflecting the chronic liver diseases were also excluded. (6) Patients having other cardiovascular diseases such as cardiomyopathy, heart valve disease, and myocarditis were excluded.

### 2.3. Clinical and Biochemical Data Collection

Clinical data such as age, gender, smoking history, diabetes mellitus history, and hypertension history were recorded from the hospital information system (HIS).

Biochemical data such as hypersensitive C-reactive protein (hs-CRP), platelet count (PLT), mean platelet volume (MPV), red blood cell (RBC), white blood cell (WBC), neutrophilic granulocyte (NEUT), red blood cell distribution width (RDW), mean corpuscular volume (MCV), hemoglobin A1c (HBA1c), fasting blood glucose (FBG), aspartate transaminase (AST), alanine transaminase (ALT), uric acid, serum creatinine (SCR), serum total bilirubin (STB), *γ*-glutamyl transpeptidase (GGT), triglyceride (TG), total cholesterol (TC), high-density lipoprotein cholesterol (HDL-C), low-density lipoprotein cholesterol (LDL-C), D-dimers, creatine kinase (CK), creatine kinase MB (CKMB), thrombin time (TT), and activated partial thromboplastin time (APTT) were recorded from the hospital central lab. The normal range of STB is from 3.4 *μ*mol/L to 17.1 *μ*mol/L by the lab protocols.

These biochemistry/hematology/coagulation tests were performed through the following instruments: Hitachi automatic biochemical immunological pipeline (LST008, E170) (Tokyo, Japan), Beckman AU5431 automatic biochemical analyzer (Ramsey, USA), Sysmex HST302 and 201 blood cell analysis automatic pipeline (Shanghai, China), and Stago STR automatic hemagglutination analyzer (Asnières sur Seine, France).

Medications at admission including aspirin, clopidogrel, angiotensin-converting enzyme inhibitor (ACEI), angiotensin receptor blockers (ARB), calcium-channel blockers (CCB), beta-blockers, and stains were recorded from the HIS.

### 2.4. The Collection of CAG Data and the Calculation of the SYNTAX Score

The patients enrolled should have the CAG results. We checked the CAG images from the catheter room and identified the clear coronary artery images for further analysis. We also checked the records of the rescue medications to confirm the safety of the patients during the CAG performance.

For calculating the SYNTAX score, several kinds of coronary lesions were considered. The calcified lesion, bifurcation lesion, main or branch lesion, and distorted lesion were included in the SYNTAX score calculation. Moreover, different weight of the coronary artery was also considered [12]. To avoid bias, two investigators separately checked the CAG images and calculated the SYNTAX score with the assistance of “SYNTAX score calculator tools” provided by the website of “www.Syntaxscore.com”. If discrepancies occurred, they would be solved through a team discussion.

### 2.5. The Collection of In-Hospital Adverse Events and 1-Year Adverse Cardiac Events of the UAP Patients

The ischemia-related events were collected from the HIS during the patient's hospitalization. Abnormal expression of hs-CRP and D-dimers, the incident of premature ventricular contraction (PVC), nonsustained ventricular tachycardia (NSVT), and atrial fibrillation (AF) were considered to have a close relationship with coronary ischemia after interventional therapies. They were checked and analyzed.

The 1-year adverse cardiac events were defined as the composite events of all-cause death, nonfatal myocardial infarction (MI), and ischemic rehospitalization. The study group collected these events through the HIS, the outpatient managing system. If necessary, telephone interviews with patients would be carried out to confirm the obscure data.

### 2.6. Statistical Analysis

SPSS19.0 statistical program package for Windows (SPSS Inc., Chicago, IL, USA) was adopted for statistical analysis. Continuous data with normal distribution were displayed as mean ± standard deviation ($\bar{X\;}\pm S$). Otherwise, they were displayed as median (interquartile). Student's *t*-test or Mann–Whitney *U* test was used for the comparison of continuous variables between two CCS-risk groups. Categorical variables were compared by the chi-square test or Fisher exact test. STB cut-off value reflecting the coronary slow flow was calculated by receive-operating curve (ROC) analysis. SYNTAX score's impact factors were identified by multivariate linear regression analysis. Cardiac events' impact factors were identified by multivariate Cox proportional hazard regression analysis. The 95% confidence interval (CI) and hazard ratios (HR) were calculated and presented. Two-tailed *P* < 0.05 was considered with statistical significance.

## 3. Results

Table 1 shows the comparisons of clinical variables between two different CCS-risk groups. Hypertension history, diabetes mellitus history, current smoking condition, and BMI had no significant differences between the two groups. However, patients in the CCS high-risk group tended to be older and more male patients. Furthermore, biochemistry indicators such as hs-CRP, MPV, NEUT, RDW, FBG, STB, LDL-C, and D-dimers were higher in the CCS high-risk group. Other indicators such as PLT, RBC, WBC, MCV, HBA1c, uric acid, SCR, ALT, AST, GGT, TG, TC, HDL-C, CKMB, TT, and APTT showed no significant differences between the two groups. More significantly, the SYNTAX score and CTFC were higher in the CCS high-risk group. They reflected the coronary conditions of the subjects. In addition to these discrepancies, medications at admission were also analyzed and showed no significant differences between the two groups.

Table 2 shows the potential variables explaining the SYNTAX score changes as analyzed by multivariable linear regression analysis. We selected variables that might have impacts on coronary arteries based on clinical experiences and differences between two coronary flow groups. Univariate analysis was primarily performed to identify the potential impact factors. Consequently, in the multivariate analysis, changes in SYNTAX score could be explained by STB (*B* = 0.59, 95% CI: 0.39-0.74, *P* < 0.01) and MPV (*B* = 0.86, 95% CI: 0.42-1.31, *P* < 0.01).

**Figure 2:**
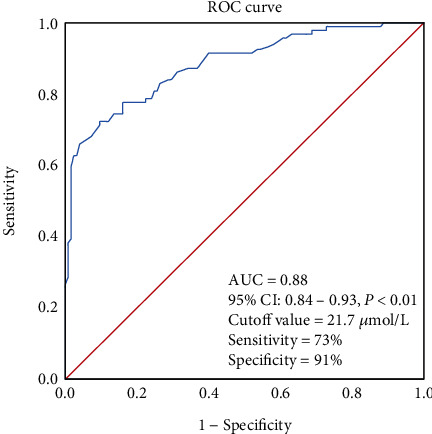
The results of ROC analysis, STB over 21.7 *μ*mol/L could reflect the coronary slow flow. The area under the curve was 0.88 (AUC = 0.88), *P* < 0.01.

Figure 2 shows the ROC results of this study. Variables potentially indicating the coronary slow flow were carefully evaluated. The highest tertile CTFC value (over 28.6 frames) was used to profile a coronary slow flow condition. STB could indicate coronary flow conditions in these subjects. The cut-off value of STB was also calculated in this section. The ROC result showed that STB over 21.7 *μ*mol/L (AUC = 0.88, 95% CI: 0.84-0.93, *P* < 0.01) could indicate a coronary slow flow condition.

To test whether STB (over 21.7 *μ*mol/L) could reflect poor clinical conditions in these patients, in-hospital coronary ischemia-related events after interventional therapy were analyzed between low-STB (STB < 21.7 *μ*mol/L, *n* = 190) and high-STB (STB ≥ 21.7 *μ*mol/L, *n* = 106) groups.  Table 3 shows that the levels of hs-CRP and D-dimers and stent numbers and the incidence of NSVT were higher in the high-STB group. The incident of AF (%) and PVC (%) showed no significant differences between the two groups.

**Figure 3:**
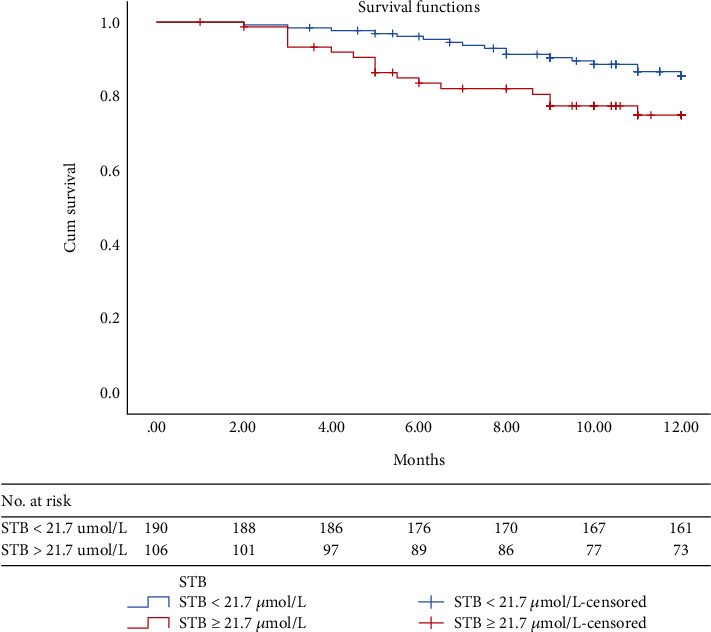
Patients with STB ≥ 21.7 *μ*mol/L had a lower event-free survival rate than patients with STB < 21.7 *μ*mol/L, and the Kaplan-Meier plots depicted the differences between the two groups of patients, log-rank *χ*^2^ = 4.75, *P* < 0.05.

Next, 1-year adverse cardiac events were collected, and a Kaplan-Meier analysis was performed (Figure 3). The rate of loss in follow-up in the STB (≥21.7 *μ*mol/L) group was 9.4% and in STB (<21.7 *μ*mol/L) group was 10.3%. Event-free survival rate was analyzed in patients with different STB levels (categorized by 21.7 *μ*mol/L). The two K-M lines began to depart from the 6th month after discharge. A deep drop of the K-M line in the high-STB line could also be found (log-rank *χ*^2^ = 4.75, *P* < 0.05).

We then performed Cox regression analysis to evaluate the impacts of STB on the 1-year outcomes of the subjects. We selected variables both with influences on coronary flow changes and clinical prognosis into the Cox analysis. Univariate analysis was primarily conducted to select the potential variables. Consequently, in the multivariate Cox HR regression analysis, STB (≥21.7 *μ*mol/L, HR = 2.01, 95% CI: 1.05-3.84, *P* < 0.01), MPV (HR = 1.22, 95% CI: 1.01-1.48, *P* < 0.01), and hs-CRP (HR = 1.29, 95% CI: 1.08-1.54, *P* < 0.01) could indicate increased 1-year poor outcomes for these patients. The data were shown in Table 4. Basic characteristics between two different STB groups after 1-year follow-up were listed in the Supplementary Table 3.

## 4. Discussions

In this study, STB elevated significantly in the CCS high-risk UAP patients, together with the elevation of several other clinical variables, such as hs-CRP, MPV, NEUT, RDW, FBG, LDL-C, and D-dimers. More significantly, elevated SYNTAX score and CTFC might reflect poor coronary conditions of the CCS high-risk subjects. As widely accepted, the SYNTAX score is a convincible clinical tool reflecting the severity of coronary lesions. We also presented that STB and MPV could explain the SYNTAX score changes of the enrolled subjects. STB is a conventional biochemistry indicator, and STB is recently more regarded as an oxidative stress-related biomarker. However, STB's roles were under debate in diseases. We in this study showed the promising indicative value of STB in UAP patients. The cut-off value of STB by ROC analysis was also presented. STB over 21.7 *μ*mol/L could indicate a coronary slow flow condition of the UAP patients. In common sense, coronary slow flow mirrors the poor condition of CAD patients [13]. We also showed that in-hospital coronary ischemia-associated events were higher in the high-STB group after interventional therapies. Confusedly, STB, a biomarker with antioxidative ability, once supposed to have vascular protective effects, is consistent with a series of coronary-related disorders in UAP patients. We tried to explain STB's indicative effects in UAP patients, which had not been discussed in articles. In this study, we also concluded that RDW, another popular biomarker, could not indicate coronary-related changes in the enrolled patients, even though it was reported as significant in CAD and CHF patients [14, 15]. In addition to STB's close relationship with coronary changes, we also verified STB's prognostic indicative effects: the Kaplan-Meier plot showed that elevated STB could indicate a reduced event-free cardiac survival rate; elevated STB with MPV and hs-CRP could indicate 1-year poor outcomes for the UAP patients. To our knowledge, until now, there still lacks a study discussing STB's close relationship with coronary flow and clinical outcomes in UAP patients.

Conditions of coronary lesions and coronary flow could determine the outcomes of CAD patients [16]. In these patients with underlying high-risk tendencies, poor prognoses could primarily be reflected by the poor coronary flow. In the clinical, UAP patients, especially the high-risk ones, often have several disorders such as hypercoagulation, platelet activation, inflammation, and oxidative stress. In recent years, it is impressive that oxidative stress and ROS contribute to the rapid progression of coronary lesions and have a close relationship with poor prognoses [17, 18]. Oxidative stress could induce an imbalance of vasodilative and vasoconstrictive cytokines and has profound impacts on coronary lesions [19]. STB is proven to be an oxidative-associated molecular [20, 21]. STB could express an antioxidative ability in a series of diseases [22]. However, in addition to being an antioxidant, STB could also reflect the detrimental effects of oxidative stress and even lead to DNA damage [23, 24]. As a result, the roles of STB in diseases were controversial. As the number of UAP patients is growing significantly these years, we noticed that STB was at relatively high levels in these patients without significant hepatobiliary disorders. We proposed that elevated STB might reflect abnormal clinical conditions for these patients. The correlations between STB and coronary changes might bring new ideas in making clinical decisions for UAP patients.

Although the vascular protective effects of STB were highlighted in recent years, low serum bilirubin levels are associated with poor clinical conditions, and STB level is negatively correlated with in-stent restenosis [25]. Low STB was once regarded as an independent risk indicator for CAD patients [26]. However, different arguments were also presented. STB's detrimental effects on patients were also discussed as previously stated. We noticed the negative correlations between STB and coronary flow in UAP patients, which had not been referred to by articles. We proposed that elevated STB in UAP patients might have similar effects as brain natriuretic peptide (BNP) in chronic heart failure (CHF) patients. In a heart failure condition, elevated BNP could either protect cardiomyocytes from heart failure attacks or indicate a severe heart failure condition.

In this study, we verified elevated STB's close relationship with the poor coronary flow in UAP patients, which might bring useful information in clinical strategies. Even though the results were clinically beneficial, a satisfying explanation for the mechanism of elevated STB in UAP patients still relies on molecular scale studies. We proposed that STB's roles in UAP patients might be interpreted by its reflection of oxidative stress burden on the subjects. STB might reflect the activation of HO-1, and HO-1 is a stress-induced enzyme. Actually, STB's poor indicative effects have already been reported in several other clinical conditions. We referenced articles and found that elevated bilirubin might indicate poor conditions in a series of diseases. Bilirubin might be a neurotoxin and induce neurologic damage [27]. STB levels are associated with a high thrombus burden in STEMI patients [28]. STB could be elevated in HF patients, and reduced STB could reflect improved end-systolic/diastolic diameters and ejection fraction (EF) after resynchronization therapy [29]. Elevated bilirubin is also an independent predictor for hypertensive patients [30]. Based on this evidence, we proposed that screening UAP patients with underlying high-risk tendencies by elevated STB might be convenient and practical.

In addition to STB's close relationship with poor coronary flow conditions, we hypothesized that elevated STB might also have a close relationship with unstable coronary plaques in UAP patients. However, this presumption needs to be verified by more clinical investigations. Being an easily acquired and gradually more promising biomarker, STB might be used to make a practical clinical decision for UAP patients without an invasive strategy. Perhaps, STB might be a risk-stratifying indicator for CAD patients in the future. This might bring new interpretations for treating these patients, especially for the ones with underlying coronary ischemia and high-risk tendencies.

### 4.1. Study Limitations

Although elevated STB showed statistical significance in this study, the collection of more cardiac events is still needed to acquire useful information in a future study. Another limitation is the consideration of Gilbert syndrome, which may interfere with STB levels by the generation of more unconjugated bilirubin (UCB). Although we excluded the patients with a jaundice history and a current jaundice status, the exclusion of Gilbert syndrome by genetic analysis could be more effective. Furthermore, the cut-off value of STB should be more precisely concluded by multicenter investigations. Moreover, the key shortcoming of this study may be due to the data from a single cardiovascular center. Multicenter collaborative analysis may induce more convincible results in the future.

## 5. Conclusions

STB levels in UAP patients might reflect changes in coronary lesions. Elevated STB (21.7 *μ*mol/L) might even indicate a coronary slow flow condition and 1-year poor outcomes for these patients.

## Figures and Tables

**Table 1:** Clinical characteristics of UAP patients between different CCS-risk groups.

Parameters	Low risk (CCS I-II, *n* = 134)	High risk (CCS III-IV, *n* = 162)	*P* value
Male (*n*, %)	86, 64.1%	121, 74.6%	<0.05
Age (years, ($\bar{X\;}\pm S$))	55.3 ± 4.7	66.5 ± 3.3	<0.01
Hypertension (*n*, %)	61, 45.5%	82, 50.6%	0.38
Diabetes mellitus (*n*, %)	29, 21.6%	31, 19.2%	0.59
Current smokers (*n*, %)	66, 49.2%	71, 43.8%	0.35
BMI (kg/cm^2^)	26.8 ± 2.6	26.5 ± 2.9	0.87
hs-CRP (mg/L)	1.1 (0.5, 2.6)	1.3 (0.6-3.3)	<0.01
PLT (^∗^10^9^/L)	195.9 ± 15.7	197.1 ± 17.6	0.43
MPV (fL)	10.8 (10.1-11.6)	11.6 (11.1-12.7)	<0.01
RBC (10^12^/L)	4.4 (3.2-5.4)	4.2 (3.5-5.9)	0.87
WBC (10^9^/L)	7.4 (5.5-9.4)	8.6 (5.7-10.5)	0.34
NEUT (%)	56.5 (44.6-77.3)	65.2 (48.5-82.1)	<0.01
RDW (%)	12.8 (12.3-13.3)	14.2 (13.6-15.7)	<0.01
MCV (fL)	93.6 (78.9-120.3)	95.7 (80.1-123.5)	0.18
FBG (mmol/L)	5.7 (5.4-6.4)	6.1 (5.5-8.3)	<0.05
HBA1c (%)	6.1 ± 1.2	6.2 ± 1.1	0.45
AST (U/L)	22.1 ± 8.9	23.1 ± 8.1	0.32
ALT (U/L)	18.2 ± 6.8	19.5 ± 7.1	0.11
Uric acid (*μ*mol/L)	220.3 (118.8-330.6)	229.2 (120.5-345.6)	0.22
SCR (*μ*mol/L)	62.3 (33.8-87.6)	64.2 (34.1-88.5)	0.15
STB (*μ*mol/L)	17.6 (15.6-20.1)	20.5 (18.3-25.5)	<0.01
GGT (U/L)	27.5 (14.5-65.4)	28.4 (17.1-63.2)	0.14
TG (mmol/L)	1.4 (0.7-3.4)	1.3 (0.9-3.2)	0.06
HDL-C (mmol/L)	1.4 ± 0.5	1.3 ± 0.6	0.12
LDL-C (mmol/L)	2.1 (1.5-2.7)	2.7 (2.2-3.1)	<0.05
D-dimers (mg/L)	0.5 ± 0.2	0.7 ± 0.3	<0.01
CKMB (IU/L)	11.5 ± 3.5	12.1 ± 4.6	0.22
TT (s)	16.5 (14.2-22.2	16.3 (13.9-23.5)	0.09
APTT (s)	30.5 (25.5-39.7)	29.6 (26.5-40.2)	0.34
SYNTAX score	23.1 ± 2.1	25.8 ± 3.6	<0.01
CTFC (frames)	24.4 (15.1-28.2)	26.4 (19.7-31.5)	<0.05
Medications at admission			
Aspirin (*n*, %)	112, 83.5%	138, 85.1%	0.71
Clopidogrel (*n*, %)	101, 75.4%	121, 74.6%	0.89
ACEI/ARB (*n*, %)	93, 69.4%	102, 62.9%	0.26
CCB (*n*, %)	88, 65.6%	103, 63.5%	0.71
Beta-blockers (*n*, %)	92, 68.6%	110, 67.9%	0.89
Stains (*n*, %)	87, 64.9%	103, 63.5%	0.81

RDW; red cell distribution width; WBC; white blood cell; MPV; mean platelet volume; ALT; alanine aminotransferase; AST; aspartate aminotransferase; CRP; C-reactive protein; HDL-C; high-density lipoprotein cholesterol; LDL-C; low-density lipoprotein cholesterol.

**Table 2:** Variables indicating the SYNTAX score changes by univariate and multivariate linear regression analyses.

Variables	Univariate analysis			Multivariate analysis			
	*B*	95% CI	*P* value	B	95% CI	*P* value	Collinearity VIF
STB	0.63	0.44-0.83	<0.01	0.59	0.39-0.74	<0.01	1.02
D-dimer	0.19	0.03-0.42	0.13				
MPV	1.14	0.73-1.45	<0.01	0.86	0.42-1.31	<0.01	1.13
RDW	0.67	0.17-1.17	<0.01				
FBG	0.01	-0.55-0.56	0.81				
hs-CRP	0.24	0.01-0.47	<0.05				
LDL-C	0.12	-0.75-1.01	0.77				

STB; serum total bilirubin; RDW; red cell distribution width; MPV; mean platelet volume; hs-CRP; high-sensitive C-reactive protein; FBG; fasting blood glucose; LDL-C; low-density lipoprotein cholesterol.

**Table 3:** Comparisons of changes after coronary interventional therapy between two different STB groups.

Variables	Low-STB (*n* = 190)	High-STB (*n* = 106)	*P* value
hs-CRP (mg/L)	1.1 (0.6-3.3)	1.2 (0.7-3.5)	<0.01
D-dimers (mg/L)	0.4 ± 0.2	0.5 ± 0.1	<0.01
Stent numbers	1.3 ± 0.3	1.7 ± 0.4	<0.05
PVC (*n*, %)	23, 12.1%	15, 14.2%	0.61
NSVT (*n*, %)	15, 7.9%	17, 16.1%	<0.05
AF (*n*, %)	13, 6.8%	7, 6.6%	0.07

hs-CRP; high-sensitive C-reactive protein; CKMB; creatine kinase MB; NT-proBNP; N-terminal-B-type natriuretic peptide; NSVT; nonsustained ventricular tachycardia; PVC; premature ventricular contraction; AF; atrial fibrillation.

**Table 4:** Variables indicating the 1-year adverse events in UAP patients by univariate and multivariate Cox proportional hazard regression analysis.

Variables	Univariate analysis			Multivariate analysis		
	HR	95% CI	*P* value	HR	95% CI	*P* value
STB (≥21.7 *μ*mol/L)	2.18	1.16-3.73	<0.05	2.01	1.05-3.84	<0.01
MPV (fL)	1.33	1.12-1.58	<0.01	1.22	1.01-1.48	<0.01
RDW (%)	1.18	0.95-1.47	0.13			
Hs-CRP (mg/L)	1.08	1.01-1.17	<0.05	1.29	1.08-1.54	<0.01
FBG (mmol/L)	1.06	0.86-1.31	0.67			
LDL-C (mmol/L)	1.14	0.86-1.51	0.43			
Age (year)	1.01	0.98-1.04	0.31			
Gender (male)	1.04	0.84-1.26	0.27			

STB; serum total bilirubin; RDW; red cell distribution width; MPV; mean platelet volume; hs-CRP; high-sensitive C-reactive protein; FBG; fasting blood glucose; LDL-C; low-density lipoprotein cholesterol.

## Data Availability

The datasets of the current study could be available from the corresponding author upon reasonable request.

## Abbreviations

SYNTAX score: The synergy between PCI with TAXUS stent and cardiac surgery score
BMI: Body mass index
hs-CRP: Highly sensitive C-reactive protein
PLT: Platelet count
MPV: Mean platelet volume
RBC: Red blood cell
NEUT: Neutrophil
RDW: Red blood cell distribution width
MCV: Mean corpuscular volume
FBG: Fasting blood glucose
AST: Aspartate transaminase
ALT: Alanine transaminase
GGT: *γ*-Glutamyl transpeptidase
STB: Serum total bilirubin.

## Supplementary Materials

Supplementary Materials A summary of the reasons for patients excluded during the screening period was listed in Supplementary Table 1. Reasons and number of patients meeting the exclusion criteria were provided. The basic features of the enrolled and excluded patients were listed in Supplementary Table 2. There were no baseline differences between the two categories of patients. We also supplied Supplementary Table 3. Characteristics between two different STB groups after one-year follow-up were listed in this table. Elevated hs-CRP, LDL-C, and MPV could still be found in the high-STB group after one-year follow-up.
