# Supplementary material for: Elevated Serum Total Bilirubin Might Indicate Poor Coronary Conditions for Unstable Angina Pectoris Patients beyond as a Cardiovascular Protector
Source: Cardiovasc Ther. 2023 Sep 5;2023:5532917. doi: 10.1155/2023/5532917 (PMC10497366; doi:10.1155/2023/5532917)
Supplement: Supplementary Materials — A summary of the reasons for patients excluded during the screening period was listed in Supplementary Table 1. Reasons and number of patients meeting the exclusion criteria were provided. The basic features of the enrolled and excluded patients were listed in Supplementary Table 2. There were no baseline differences between the two categories of patients. We also supplied Supplementary Table 3. Characteristics between two different STB groups after one-year follow-up were listed in this table. Elevated hs-CRP, LDL-C, and MPV could still be found in the high-STB group after one-year follow-up. [file 5532917.f1.docx]

| **Supplementary Table1, a summary of patients excluded during the screening process** | | |
| --- | --- | --- |
| **Reasons for exclusion** | **Total number 824** | **%** |
| **Patients over 80 years old** | **56** | **6.8** |
| **UAP patients with contradictions for CAG** | **291** | **35.3** |
| Low platelet numbers (≤20*10^9^) | 27 | 3.3 |
| Severe anemia (HB≤60g/L） | 20 | 2.4 |
| Combined with pneumonia | 35 | 4.2 |
| Combined with acute bronchitis | 34 | 4.1 |
| Combined with acute pharyngitis | 36 | 4.4 |
| Combined with acute swelling arthritis | 24 | 2.9 |
| Combined with acute diarrhea | 7 | 0.8 |
| Combined with gout | 47 | 5.7 |
| Ischemic stroke within 3 months | 12 | 1.5 |
| Cerebral hemorrhage with 6 months | 8 | 1.0 |
| With uncontrolled blood glucose (FBG ≥11.1mmol/L) | 12 | 1.5 |
| With poor BP (SBP≥160 mmHg, or DBP≥100mmHg) | 9 | 1.1 |
| With coagulation disorders |  |  |
| TT over 21 S | 15 | 1.8 |
| APTT over 53 S | 5 | 0.6 |
| **With factors affecting STB levels** | **348** | **42.2** |
| Chronic hepatitis B | 48 | 5.8 |
| Chronic cholecystitis | 37 | 4.5 |
| Chronic pancreatitis | 24 | 2.9 |
| With jaundice history | 65 | 7.9 |
| Abnormal ALT(≥80U/L) | 74 | 9.0 |
| Abnormal AST(≥80U/L) | 55 | 6.7 |
| Abnormal ALP(≥220U/L) | 45 | 5.5 |
| **Combined with other cardiovascular diseases** | **85** | **10.3** |
| Acute carditis | 13 | 1.6 |
| Moderate to severe Mitral restenosis | 35 | 4.2 |
| Aortic valve stenosis | 22 | 2.7 |
| Combined with pulmonary embolism | 15 | 1.8 |
| **Patients unwilling to share their data** | **44** | **5.3** |

UAP, unstable angina pectoris; CAG, coronary angiography; HB, hemoglobin; FBG, fasting blood glucose; BP, blood pressure; TT, thrombin time; STB, serum total bilirubin; AST, aspartate aminotransferase; ALT, alanine aminotransferase; APTT, activated partial thromboplastin time; ALT, alanine transaminase.

| Supplementary Table 2, a comparison of basic features between the enrolled and excluded patients | | | |
| --- | --- | --- | --- |
| variables | **Enrolled patients**  **(*n* = 296)** | **Excluded patients**  **(*n* = 824)** | ***p-value*** |
| Age (years，($\bar{\boldsymbol{X}}\boldsymbol{\pm}\boldsymbol{S}$) ) | 61.2±6.8 | 60.5±5.9 | 0.11 |
| Hypertension (n, %) | 143, 48.3% | 418, 50.7% | 0.27 |
| Diabetes mellitus (n, %) | 60, 20.7% | 173, 21.0% | 0.79 |
| Current smokers (n, %) | 137, 46.2% | 388, 47.1% | 0.81 |
| BMI (kg/cm2) | 26.7±2.6 | 26.9±2.9 | *0.27* |

BMI, body mass index.

| Supplementary Table 3, characteristics between two different STB groups after 1-year follow-up from the HIS. | | | |
| --- | --- | --- | --- |
| Variables | **low-STB (n=172)** | **high-STB (n=95)** | ***P-value*** |
| hs-CRP (mg/L) | 0.9(0.5-2.4) | 1.3(0.6-2.9) | <0.05 |
| D-Dimers (mg/L) | 0.3±0.1 | 0.4±0.1 | 0.46 |
| LDL-C (mmol/L) | 2.3± 0.4 | 2.5± 0.3 | <0.05 |
| STB (μmol/L) | 16.9 (12.1-19.6) | 23.6 (21.6-26.4) | <0.01 |
| MPV (fL) | 11.8(9.1-13.5) | 12.1(10.3-13.7) | <0.05 |
| Medications at 1-year follow up |  |  |  |
| Aspirin (n, %) | 165, 95.9% | 87, 91.6% | 0.33 |
| Clopidogrel (n, %) | 157, 91.2% | 86, 90.5% | 0.84 |
| ACEI/ARB (n, %) | 142, 82.5% | 76, 80.0% | 0.80 |
| CCB (n, %) | 96, 55.8% | 55, 57.8% | 0.63 |
| Beta-blockers (n, %) | 125, 72.6% | 70, 73.6% | 0.69 |
| Stains (n, %) | 169, 98.2% | 90, 94.7% | 0.37 |

hsCRP, high-sensitive C-reactive protein; LDL-C, low density lipoprotein cholesterol; STB, serum total bilirubin; MPV, mean platelet volume; ACEI, angiotensin converting enzyme inhibitor; ARB; angiotensin recenter blocker; CCB, calcium channel blocker.
